# High-Resolution Description of Antibody Heavy-Chain Repertoires in Humans

**DOI:** 10.1371/journal.pone.0022365

**Published:** 2011-08-04

**Authors:** Ramy Arnaout, William Lee, Patrick Cahill, Tracey Honan, Todd Sparrow, Michael Weiand, Chad Nusbaum, Klaus Rajewsky, Sergei B. Koralov

**Affiliations:** 1 Department of Pathology, Brigham and Women's Hospital, Boston, Massachusetts, United States of America; 2 Genome Sequencing and Analysis Program, Broad Institute of MIT and Harvard, Cambridge, Massachusetts, United States of America; 3 Program in Cellular and Molecular Medicine, Children's Hospital and Immune Disease Institute, Harvard Medical School, Boston, Massachusetts, United States of America; Innsbruck Medical University, Austria

## Abstract

Antibodies' protective, pathological, and therapeutic properties result from their considerable diversity. This diversity is almost limitless in potential, but actual diversity is still poorly understood. Here we use deep sequencing to characterize the diversity of the heavy-chain CDR3 region, the most important contributor to antibody binding specificity, and the constituent V, D, and J segments that comprise it. We find that, during the stepwise D-J and then V-DJ recombination events, the choice of D and J segments exert some bias on each other; however, we find the choice of the V segment is essentially independent of both. V, D, and J segments are utilized with different frequencies, resulting in a highly skewed representation of VDJ combinations in the repertoire. Nevertheless, the pattern of segment usage was almost identical between two different individuals. The pattern of V, D, and J segment usage and recombination was insufficient to explain overlap that was observed between the two individuals' CDR3 repertoires. Finally, we find that while there are a near-infinite number of heavy-chain CDR3s in principle, there are about 3–9 million in the blood of an adult human being.

## Introduction

The human adaptive immune system consists of B cells, which secrete antibodies, and T cells, which target infected or otherwise aberrant cells through their T cell receptors (TCRs). Both cell types have been of longstanding interest for their roles in vaccines, infections, and autoimmune diseases, as well as cancer [1], [2]. Underlying these roles is the ability of B and T cells to generate a seemingly infinite number of different antigen specificities from the finite genetic material encoded in the germline genome.

Several mechanisms are responsible for generating this diversity. The most fundamental is somatic recombination [3]. This is a combinatorial process in which any one of several gene segments from each of two or three sets of segments are recombined to form a single novel gene (a highly ordered and regulated process). Each antibody molecule is made up of the protein products of two such genes, called heavy chain and light chain. The heavy-chain gene is constructed, through somatic recombination, of three gene segments, called V, D, and J; there are 56 V, 23 D, and 6 J segments in the human genome [4]. The sequence from the V-D to the D-J junction, spanning the entire D segment, is called complementarity determining region (CDR) 3 and encodes part of the heavy chain that makes physical contact with the antigen. It is the single most important determinant of an antibody's antigen specificity [5], [6]. Hence detailed descriptions of CDR3 diversity are a prerequisite for understanding antibody responses to vaccines and infections and in autoimmunity in fine detail—the level of detail required for rational approaches to development of the next generation of diagnostics and therapeutics [1], [2].

There has been growing interest in using high throughput sequencing for describing antibodies [7], [8] and TCR [9], [10], [11], [12]. Recent studies have used sequencing to describe the antibody repertoire in zebrafish [13], to estimate the diversity of TCR in humans [12], and to monitor residual disease in leukemia patients [14]. The B cell repertoire in humans and mice has been the subject of a number of detailed studies, especially of antibody responses to various diseases, but not typically at the scale offered by high-throughput sequencing [15], [16], [17], [18], [19], [20], [21].

Since the diversity of antibody sequences depends on VDJ recombination, a number of previous studies have investigated the diversity of VDJ joints expressed in response to specific infections, malignancies, and autoimmune diseases [15], [16], [17], [18], [19], [20], [21]. They showed that for many exposures, genetically different individuals produce antibodies with the same heavy- or light-chain V(D)J combinations [22], [23], [24], [25]. This has been observed most often in antibody responses to bacterial polysaccharide antigens, which are of interest because they are the targets of protective and vaccine responses against infections by a number of medically important life-threatening bacteria, such as *Haemophilus influenzae* and *Streptococcus pneumoniae* [26]. One of the best-known examples is the response to the childhood vaccine *for H. influenza* type B, in which protective antibodies use V segment V_H_3-23, either J segment J_H_4 or J_H_6, and a D that results in a heavy-chain CDR3 that contains the protein sequence GYGF/MD [26]. Studies have shown the existence of so-called public sequences [27] (overlap among the repertoires of different individuals), an unlikely occurrence if repertoires are shaped exclusively by chance.

In this context, we sought to describe the baseline diversity of V, D, J, and CDR3 repertoires in antibody heavy-chain genes in human and mouse using high-throughput sequencing with particular attention to the roles of somatic recombination and positive and negative selection.

## Materials and Methods

Using 454 sequencing [28], we sequenced antibody heavy-chain VDJ-rearranged genes from two healthy, genetically unrelated young adult male donors. We used variants we developed of primers that are well known from clinical laboratory medicine for diagnosing clonal rearrangements in human leukemias [29]. We also sequenced VDJ-rearranged genes from pooled spleens from C57BL/6 mice to begin to understand which characteristics of these immunomes are specific to humans and which apply also to this model system.

### Sample collection

For human, PBMC were isolated from 4×10 ml of peripheral blood drawn from one male donor and 2×10 ml from a second male donor using BD Vacutainer CPT cell preparation tubes (BD) in compliance with the institutional review board at our institution (protocol number 0510001399 of the Brigham and Women's Hospital Committee on the Use of Humans as Experimental Subjects, Brigham and Women's Hospital, Boston, MA; this protocol was considered exempt after review by the Committee pursuant to Federal regulations, 45 CFR Part 46.101(b)(4)). For mouse, total spleen was collected from 4 C57BL/6 mice and B cells isolated magnetically (MidiMACS B Cell Multisort Kit II; Miltenyi). For both species, total DNA was then isolated by standard methods (Qiagen). Mice were housed and cared for under specific pathogen-free conditions in accordance with protocol number 03341, approved by Harvard Medical School IACUC guidelines.

### Amplification and sequencing

Recombined heavy chain fragments were amplified from DNA obtained as above using a subset of immunoglobulin V_H_ FR2 (VH1-FR2, VH2-FR2, VH3-FR2, VH5-FR2, and VH6-FR2; VH4-FR2 and VH7-FR2 had redundant coverage) or FR3 (VH1-FR3 through VH6-FR3 but not VH7, which was redundant with VH1) and J_H_ BIOMED-2 primers [29] for human and a multiplex cocktail of four forward primers with homology to heavy-chain FR3 (5′-AAGTTCAAGGGCAAGGCC-3′, 5′-CTCCAAGAGCCAAGTTTTCTT-3′, 5′-CAATCTCCAAGGATACCTCCA-3′, 5′-CGITTCACCATCTCCAGAGA-3′) and two reverse primers with homology to J consensus (5′-CTTACCTGAGGAGACGGTGAC-3′ and P-AGGACTCACCTGAGGAGAC-3′) for mouse. All samples were amplified by PCR for 35 cycles with PlatinumTaq (Invitrogen) with the following conditions: 1.5 mM MgCl2; melt at 95°C for 1′00″, anneal at 60°C for 1′00″, extend at 72°C for 1′30″ and then sequenced by 454 sequencing.

### Primer design

Primers were designed using an approach developed in house and designed to attain maximum coverage of a heterogeneous set of target sequences with a minimal, non-self-hybridizing primer set. Briefly, for each set of targets, we made a list of all primer-length substrings with low predicted self-hybridization. We then evaluated the number of targets “hit” by each substring, allowing up to three (non-terminal) mismatches. The substring with the most hits was added to the primer set. The process was repeated for the remaining targets until we had a set of primers that hit all targets. We then added, removed, or changed single nucleotides to achieve similar melting temperatures. V segments identical over the sequenced region were placed into equivalence classes.

### Sequence assignment

V, D, and J segments were assigned and aligned to sequences through a two-step method. In the first step, the segment with the most substrings in common with the sequence was assigned to that segment. The longer the segment, the better this kind of approach does; therefore we expected the highest accuracy in assigning V segments, which are longest, followed by J, then D. The V segment was assigned first, followed by J, then D; we required that V and J be at opposite ends of the sequence and D be between them. (D regions, which are short, cannot be reliably assigned in sequences with high somatic hypermutation.) When multiple segments tied for having the same number of substrings in common with the sequence, the sequence was marked as an ambiguous assignment and was removed from subsequent analysis. In the second step, megablast (v2.2.23) was used to align the assigned segment to the sequence.

For analysis of individual recombination events, we then used a substring comparison similar to step 1 to group sequences into clusters [13], and chose a representative sequence for VDJ analysis. This alignment provided coordinates from which chewbacks and N- or P-nucleotide additions were calculated. Following earlier reports [30], sequences with V and J chewbacks of ≥30 nucleotides were removed from analysis.

### Validation of annotation

We validated our approach on synthetic data sets consisting of tens of thousands of VDJ-recombined sequences that we produced *in silico*. For each synthetic data set, the number of bases removed by exonucleotide chewback, added by nontemplated nucleotide insertion, and mutated by somatic hypermutation was chosen according to previous reports [23], [31], [32], [33], [34], [35], [36]. Our method correctly assigned 97–99% of V segments and 91–98% of J segments in both human and mouse synthetic data sets; it was incorrect for <1% of these segments (<1% false positives). It correctly assigned 65–67% of D segments in human but only 33% in mouse, in which D segments are much shorter than in human; it incorrectly assigned 5–10% of D segments. It is worth noting that in most cases in which the D segment was incorrectly assigned, the reason was the presence of mutations that made the sequence more similar to a different D segment—that is, objectively speaking, the incorrect assignment was a better match than the correct assignment would have been. This is a consequence of D segments being short and similar to each other in sequence.

### Statistical analysis

In addition to basic statistics about the frequencies of each V, D, and J, we investigated the statistical likelihood of preferential pairing between segments (e.g., a given D with a given J). We did this as follows.

The likelihood of preferential pairing between two segments *i* and *j* was calculated for each pair by calculating the probability of observing n instances of an *ij* pair given the frequencies of *i* and *j* among all observations. For example, consider a hypothetical genome with only two different V segments, V1 and V2, and two different D segments, D1 and D2. Suppose we were to find that V1 and V2 each appeared in half of 100,000 V-D recombined sequences, as did D1 and D2. If V-D pairing were random, one would expect the combinations V1-D1, V1-D2, V2-D1, and V2-D2 should each appear 25% of the time, or in 25,000 sequences each. But suppose the data show V1-D1 and V2-D2 in 50,000 sequences each and V1-D2 and V2-D1 in zero sequences. The probability of observing 50,000 V1-D1 or V2-D2 sequences by chance, given that the segments each appear half the time, is very small; this suggests preferential pairing between V1-D1 and V2-D2.

To determine if the probabilities at which each VD, DJ, and VJ pair appeared in our data were statistically significant, we compared these probabilities to the probabilities that we observed in hundreds of *in silico* simulations. In each simulation, we constructed 20,000–70,000 antibody sequences at random using the same individual frequencies of V, D, and J segments observed in our real data sets (the number of sequences chosen to be the same as the number in our real data sets), assigned V, D, and J, and recorded the frequencies at which each VD, DJ, and VJ pair appeared in the simulation. In these simulations, in tens of thousands of measurements of the frequencies of segment pairs, 95% of the calculated probabilities were p>0.1, and the minimum probability observed was p = 10^−4.5^ ( = 3.2×10^−5^). Therefore, in our real data, we considered probabilities less than 10^−4.5^ to be statistically significant—i.e., less than what was observed by chance. Preferential pairing was always confirmed in both directions: for example, if V1 was found in twice as many D1-containing sequences as expected, we confirmed that D1 was also found in twice as many V1-containing sequences as expected.

## Results

### V, D, and J gene segments, as well as VDJ combinations, appear at unequal frequencies in humans

We obtained 746,248 reads from a single human subject: 453,223 reads from FR3 to the consensus J region and 293,025 reads from FR2 to the consensus J region (Table 1). V segments that were identical over these regions were combined into equivalence classes. This process led to the identification of 52,011 clusters from the FR3-to-consensus-J reads and 14,848 clusters from the FR2 to consensus J reads, each cluster representing a single VDJ recombination event.

**Table 1:** Number of Reads and Clusters.

	Subject 1		Subject 2
	FR2 to J	FR3 to J	FR3 to J
VJ reads	293,025	453,223	66,557
Clusters	14,848	52,011	6,226
*Unique V and J*	*8,274*	*32,986*	*3,479*
*Unique V, D, and J*	*7,041*	*29,240*	*2,737*
Average reads/cluster	19.7	8.7	10.7

**Figure 1:**
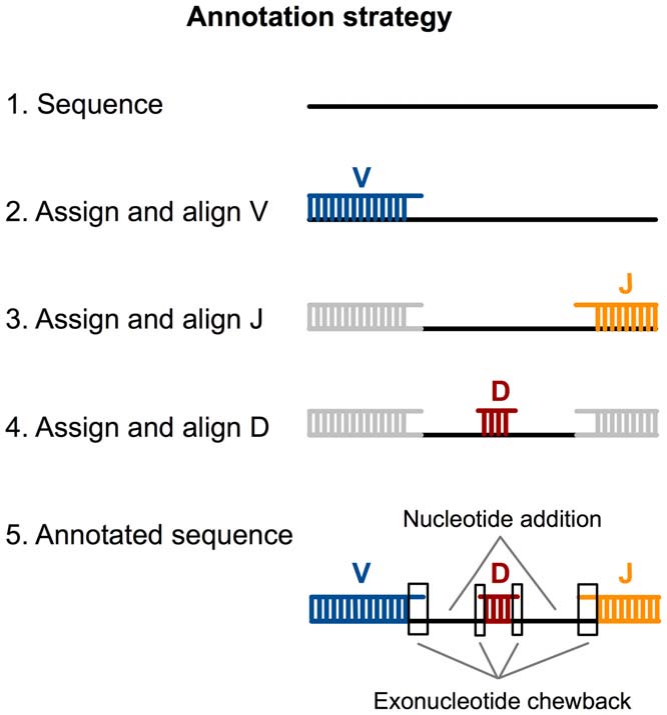
A five-step annotation methodology was used. Step 1: New sequence was obtained through pyrosequencing. Step 2: Using a word-counting strategy, the best-match V was determined. When there was a tie for best match, or when the best match was nevertheless a poor match, sequences were removed from subsequent V-D-J usage analysis. The best-match V was then aligned to the new sequence using megablast. Steps 3: The region that matched V was removed (grayed out in the figure) and the same word-counting strategy was applied to the remaining sequence to determine the best-match J sequence. As with V, ties and poor matches were removed; the best-match J was aligned using megablast. Step 4: The region that matched J was removed (gray) and D was assigned and aligned to the remainder as in Steps 2 and 3. Step 5: Using the alignments, exonucleotide chewback and nucleotide addition were determined.

**Figure 2:**
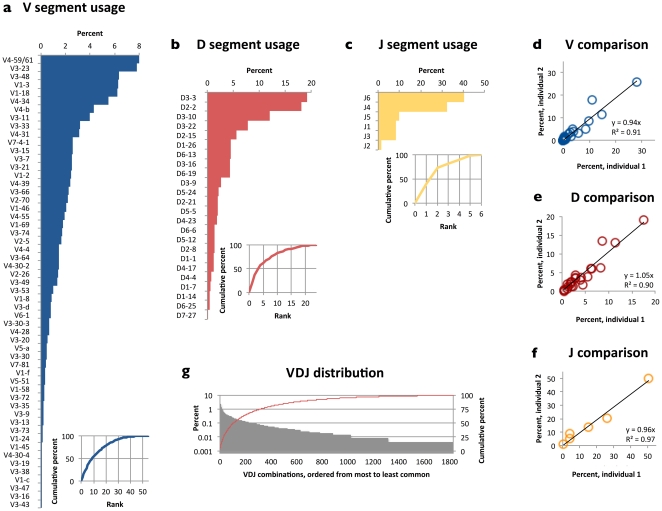
V, D, and J segment use in human is highly uneven. Lines indicate cumulative distributions (measured on right-hand side y-axis) (a)–(c). Not all V segments are labeled on the x-axis. V, D, and J segments appear at similar frequencies in two different human subjects (d)–(f). Each point corresponds to a single segment. Insets show cumulative distributions. VDJ combinations also appear unevenly, with the 100 most frequent VDJ combinations responsible for 50 percent of all recombination events (g).

We annotated sequences according to the V, D (where possible), and J segments that comprised them (Fig. 1). V, D, and J gene segments appeared at unequal frequencies, with a few segments appearing very often and the majority appearing rarely, resulting in highly skewed or long-tail distributions (Fig. 2a–c). The most frequently observed V regions were V_H_4-59/61 (the VA equivalence class), V_H_3-23, and V_H_3-48, consistent with previous reports [7], [37]. Only 10 V regions accounted for half of all clusters, underscoring the long tail in Fig. 1a (oligoclonality index [OI] = 0.56 [38]). A similar pattern was observed from D regions, with four of the 23 D segments—IGHD3-3, D3-10, D2-2, and D3-22—accounting for half of all Ds (OI = 0.60). The long-tail pattern was least pronounced for J segments, since there are only six of them, but J4 and J6 were by far the most frequently used, consistent with prior reports [39] (OI = 0.55). This pattern was identical between the two sequenced individuals (Fig. 2d–g) with an average difference of 1.1% across all segments.

Because V, D, and J segments appeared at unequal frequencies, we expected that unique VDJ combinations, which are the product of the segments, would appear at unequal frequencies as well. Indeed, frequencies ranged from <0.1% to 2.0% for the 1,829 different VDJ combinations observed in the FR3-to-J set and 0.1% to 2.7% for 1,699 different combinations observed in the FR2-to-J set, again with long tails (Fig. 2g). However, other factors might also contribute to unequal frequencies of VDJ combinations, including preferential pairing between segments or positive or negative selection of B cells based on the VDJ combination that each one harbors. We investigated each of these possibilities in turn.

### V, D, and J segments show minimal preferential pairing

We asked whether DJ, VD, and VJ segment pairings exhibit preferential pairing—that is, whether particular pairs appear more often or less often than one would expect by chance. During somatic recombination, D and J segments recombine first. We found that 16 of 186, or 8.6%, of observed DJ pairs exhibited preferential pairing, for example, the D segment D3-9 and the J segment J3. Because D3-9 was observed in 2.9% of all sequences, it was expected to appear in 2.9% of sequences that contained J3. However, we found that D3-9 appeared in 6.0% of J3-containing sequences (p = 1.1×10^−6^), or 6.0/2.9 = 2.1-fold preferential pairing. Thus D3-9 and J3 pair about twice as often as expected by chance. As another example, D3-22 was expected to occur in 8.2% of J6-containing combinations, but was observed in just 3.8% of them, less than half as often as expected by chance (p = 2.9×10^−45^). Thus, D3-22 and J6 also exhibit preferential pairing, though in this case it is a 2–3-fold *negative* preference (a preference *against* pairing with each other). The remaining 14 DJ pairs exhibited preferences somewhere between these values.

V-DJ recombination follows D-J recombination. In contrast to DJ pairs, only 0.3% (2 of 768) of VD associations showed preferential pairing. Because D segments are very short—half have fewer than 20 nucleotides—and since they lose nucleotides due to chewback during recombination, we also looked for preferences among VJ pairs, on the hypothesis that a J segment's sequence or flanking sequences could influence the choice of V (even though V and J almost never [40] pair directly). Again, the effect was small: just 1.3% of VJ pairs showed preferential pairing. These observations were nearly identical between the two individuals. There was no obvious correlation between the extent of preferential pairing and the amount of microhomology [41], [42], [43] between segments or the sequence of their recognition signal sequences (RSSes; not shown). Thus the preferential pairing that occurs among segments in VDJ recombination is almost all during the first step of recombination, between D and J.

### VDJ combinations appear at similar frequencies in productive and nonproductive joins

We next asked whether these preferences and the frequencies of VDJ combinations were determined during recombination, or reflected subsequent positive or negative selection on B cells expressing the resulting antibodies. To test whether selection determines the frequency of VDJ combinations, we compared the frequencies of VDJ combinations in productive vs. nonproductive combinations. We considered nonproductive those rearrangements that are either out of frame or contain stop codons. Because nonproductive rearrangements are not expressed, they are not under selection; thus differences in the frequency of a given VDJ between productive and nonproductive rearrangements would suggest that cells are selected for or against based on the VDJ combination.

We calculated the difference in frequency for each combination and, using synthetic data sets as benchmarks (see Methods), the probability that this difference would be seen by chance. By this approach, only 2% of VDJ combinations showed a statistically significant difference between productive and nonproductive rearrangements. In fact, we found *less* difference between productive and nonproductive combinations in the actual data set than in our benchmark sets, in which productive and nonproductive combinations are produced at random. Taken together, these findings suggest that while selection for antigen specificity is known to be important, VDJ combination alone does not determine antigen specificity, in clinically healthy individuals.

### Segment usage, pairwise preferences, and selection on VDJ combinations in mice

**Figure 3:**
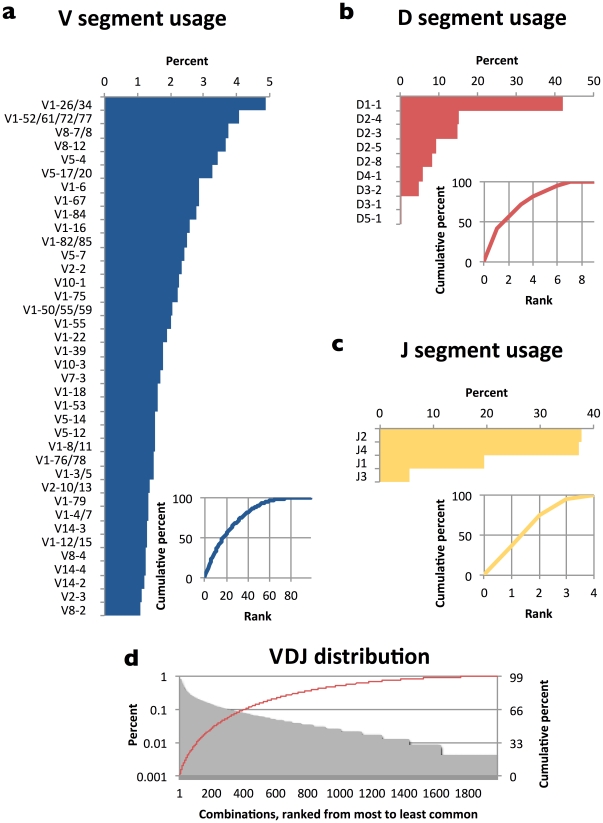
V, D, and J segment use in mouse is also highly uneven. Lines indicate cumulative distributions (measured on right-hand side y-axis) (a)–(c). Out of space considerations only V segments present at ≥1% are shown. Insets show cumulative distributions. VDJ combinations also appear unevenly, with the ∼200 most frequent VDJ combinations responsible for 50 percent of all recombination events (d).

We conducted the same analysis on VD, DJ, and VJ pairs, and VDJ combinations, from C57BL/6 mice. These mice have 100 V, 10 D, and 4 J functional gene segments [44]. As in human, we found “long tail” distributions for V, D, and J segment usage and in VDJ combinations (OI = 0.56, 0.52, and 0.68 for V, D, and J respectively; Fig. 3a–d). Using the same methodology as above, we found that 27.6% of DJ pairs showed statistically significant preferential pairing—higher than in human—while VD and VJ pairs showed almost no statistically significant preferential pairing (0.0% and 1.1%, respectively). For example, D3-2 appeared in 19.4% of sequences overall, but in only 2.2% of J1-containing sequences, an 8.7-fold negative preference (p = 2×10^−72^). Meanwhile, J3 appeared in 15.0% of all sequences but 20.7% of sequences that contain D2-4, a 40% positive preference (p = 5×10^−8^). Most preferences were less than 2-fold. As in human, there were no significant differences between productive and nonproductive combinations, suggesting that all VDJ (and also VD, DJ, and VJ) combinations, once formed, are equally able to contribute to functional antibodies.

### Deep sequencing confirms overlap among CDR3 sequences between human subjects

Next, we inquired about similarities between the two human individuals. There are approximately one billion B cells in the human body, 10 million of which are produced and destroyed each day. Consequently, a single individual can only ever express a tiny fraction of the potential CDR3 repertoire. Yet CDR3s with the same amino acid sequences have been observed in different individuals, in antibodies and TCR [12], [45]. We sought to quantitate this for antibody heavy-chain CDR3s, and asked whether it could happen by chance.

To do this, we built synthetic data sets with the same frequencies of V, D, and J segments, the same chewback and N-nucleotide addition profiles, comparable mutation rates, and the same number of sequences, as our real data sets. Unsurprisingly, we found that the number of sequences in common depends greatly on the mutation rate used to create the data sets: the higher the mutation rate, the fewer sequences in common between two sets. For example, with a mutation rate of 0.05 (an average of one in every 20 bases mutated per sequence), in 585 pairwise comparisons among 54 synthetic data sets, we found an average of 236 CDR3s in common (s.d., 15). Raising the mutation rate to 0.10, in 1,000 pairwise comparisons among 110 synthetic data sets, we found an average of 75 CDR3s in common (s.d., 8). At the other extreme, with a mutation rate of zero, the overlap was 1,292 (s.d., 34). Meanwhile, in actual data from our two human subjects, we found 279 CDR3s in common. We estimated the mutation rate in our actual data sets as between 0.05 and 0.08, based on comparing annotation with synthetic data sets that had varying mutation rates (sequencing error makes precise determination of mutation rate unreliable at this depth of sequencing). Thus although the overlap between human subjects was only a fraction of the total CDR3 diversity, it was significantly more than was expected by chance (p = 0.002 for comparison with a mutation rate of 0.05; p<<0.0001 for comparison to a mutation rate of 0.10).

Statistically, it is more likely that two short CDR3s will have the same sequence than two longer ones (in the limit, two CDR3s with a length of just a single amino acid would have at least a 1∶20, or 5%, chance of overlap because there are 20 amino acids). Therefore we expected the CDR3s that were shared between synthetic data sets, as well as the CDR3s shared between our two human subjects, would be shorter than CDR3s overall. In the synthetic data sets, the lengths of shared CDR3s again depended on mutation rate. For a mutation rate of 0.05, the shared CDR3s were less than half as long as CDR3s overall: they had a modal length of 7 amino acids (mean±s.d., 9.9±3.4; n = 141,121) vs. a modal length of 15 for CDR3s overall (15.0±3.8; n>3.1 million). The length of the shared CDR3s decreased as the mutation rate increased. In contrast, in the actual data from our human subjects, shared CDR3s were only slightly shorter than the overall average: 12 (14.3±3.7; n = 279) vs. ∼15 amino acids in each of the two data sets (15.6±3.8 and 15.3±3.6; n = 189,965 and 24,979, respectively). The CDR3s shared by the two human subjects were significantly longer than the shared CDR3s in the synthetic data sets (12 vs. 7 amino acids; V, D, and J segment usage was similar in both shared sets). Thus both the degree of overlap and the length of the shared CDR3s were greater than expected by chance, arguing that the overlap in CDR3 sequence among individuals is biological and not a statistical artifact. CDR3s in the human data sets also shared other properties not seen in the synthetic data sets. Amino acid charge was −0.05±1.40 and 0.09±1.39 for productive joins and 1.12±1.55 and 1.10±1.51 for nonproductive joins in the two individuals. These differed from charge properties in the synthetic data sets (−0.39±1.17 for productive and 0.74±1.28 for nonproductive representative synthetic datasets), suggesting selection for CDR3s that carry a slightly more positive charge.

### Estimate of total antibody heavy-chain CDR3 diversity in human

Finally, deep sequencing allowed us to estimate the total number of productive heavy-chain CDR3s in each of our two human subjects. We did this as follows. In the first subject, the first 1,000 FR3-to-J sequences that we obtained contained 953 unique productive CDR3s. The next set of 1,000 added another 885 unique productive CDR3s; the third set added another 831, and so on, in a pattern of logarithmic decay (R^2^ = 0.98; not shown). In general, each subsequent set added fewer unique CDR3s. The same pattern was observed in the FR2-to-J sequences, and in the second subject. Fitting logarithmic curves to these trends allowed us to calculate, for each sample, the point at which no additional sequences would be observed. This produced an estimate of 48,000–69,000 unique productive heavy-chain CDR3s from samples from the first subject and 12,500 unique productive heavy-chain CDR3s from samples from the second subject. Because the number of CDR3s in common between two 10-ml samples from the same subject was small relative to the total number of CDR3s in each sample (not shown), assuming the body has 5 liters of blood, we multiplied to get an upper bound of 3 to 9 million heavy-chain CDR3s in the blood of an adult human.

## Discussion

The specificity of adaptive immune responses is encoded in the sequences of recombined antibody and TCR genes. This makes sequencing a useful modality for dissecting immune responses, as has long been recognized. Since the first studies on mice over two decades ago [23], [31], [46], a central question has been how similar are immune responses between different individuals. This is related to another long-standing question: what is the actual—as opposed to the potential or theoretical [7]—diversity of the adaptive immune response in humans? The answers bear on the feasibility of developing sequenced-based diagnostics to monitor specific immune responses in vaccination, infection, cancer, and autoimmunity, and the general state of the immune system in immunodeficiency states like immunosuppression and aging.

Here we show just how important the first step in generating diversity—the choice of V, D, and J segments during somatic recombination—is for determining antibody diversity. We find that in humans the VDJ repertoire is almost entirely set by whatever genetic controls determine the selection of one V, D, or J segment over another, with only a small bias of preferential pairing of certain Ds with certain Js. The most frequently used Vs and Ds that we observed were the same as those observed in previous single-cell sequencing studies [39]. The scale of those studies was too small to provide accurate or precise statistics on all segments to compare with our results, but was sufficient to provide statistics on the frequencies of V and D families, and also the six J segments. We found more preferential pairing in mouse than in human, again limited to DJ pairs. But on the whole, the unequal frequencies at which heavy chains with different VDJ combinations appear depend on the unequal frequencies at which their component segments are chosen for recombination—and not on positive or negative selection acting on cells that express certain VDJ combinations and not others. We note that the long-tail distributions we observed for V and D segments corroborate previous reports [39] and are similar to what was seen for TCR beta-chain V-region usage [47]. At a genetic level, recombination depends on recombination signal sequences (RSSs) associated with each segment [3]. While it is known that variations in the canonical RSS can decrease recombination efficiency [48], and thus decrease the frequency at which a given gene segment is chosen for recombination, we observed no precise correspondence between RSS sequence and gene segment frequency.

Previous studies found that the overlap in TCR CDR3s among mice resulted from the uneven usage of gene segments [11]. While uneven usage is necessary to explain the overlap of CDR3s observed between our two human subjects, we show that it is not sufficient. Controlling for V, D, and J usage, exonucleotide chewback and N-nucleotide addition, and (as much as possible) mutation rate, we nevertheless observed significantly more overlap than was expected by chance, reminiscent of the unexpectedly high overlap among TCR observed in recent studies [12]. Also, the lengths of the overlapping CDR3s differed substantially from the lengths of overlapping CDR3s in synthetic data sets, and was much closer to the length profile of CDR3s overall, further suggesting that the observed overlap was not simply a function of V, D, and J segment recombination. The segment usage, chewback, nucleotide addition, and mutation rate we used in our synthetic data sets amount to a model of antibody heavy-chain gene formation; the observed overlap is not explained by our model. While it is conceivable that controlling for other statistical features in our model could make our synthetic data sets closer approximations of real ones, we consider it unlikely that it would make enough of a difference to substantially increase the number of CDR3s in common between synthetic data sets, or double their length. The alternative explanation is convergence based on biological factors, including positive selection for B cells that express antibodies with these heavy-chain CDR3s.

Our estimate of total antibody heavy-chain CDR3 diversity is roughly the same order of magnitude as estimates of heavy-chain TCR-beta diversity [10], [47]. However, many questions remain. First, although heavy-chain CDR3 is the most important determinant of antigen specificity, it is not the only one. Sequence variation in CDR1 and CDR2 remain to be investigated, as does the larger issue of patterns of pairing between heavy chain and light chain. Combinatorial libraries built from human heavy- and light-chain sequences have been produced [7], but the diversity of heavy-light chain pairs in humans *in vivo*, as opposed to in synthetic libraries [37], [49], has not yet been directly characterized. Second, the frequency of each CDR3—meaning the number of B cells, clonally related, that express that CDR3—remains uncertain, since PCR amplification can introduce biases in quantitation. Third, variation among functional subsets of B cells, such as naïve and memory B cells and plasma cells, as well as antigen-specific B cells, in different states of health and disease, has yet to be investigated in similarly fine detail. However, our technique and results help establish a basis with which to design immune sequencing studies to address these and other questions.
